# Metabolic syndrome in psychiatric patients with schizophrenia

**DOI:** 10.1192/j.eurpsy.2024.1525

**Published:** 2024-08-27

**Authors:** I. Bakija, M. Tripković, S. Kaštelan, M. Bogadi, V. Grošić

**Affiliations:** ^1^Department of Integrative Psychiatry, Psychiatry Clinic Sveti Ivan; ^2^Department of Psychiatry and Psychological Medicine, University Hospital Centre Zagreb; ^3^Department of ophthalmology, Clinical Hospital Dubrava; ^4^Psychiatric Hospital for children and adolescents, Zagreb, Croatia

## Abstract

**Introduction:**

Metabolic syndrome and cardiovascular diseases are a very important cause of morbidity and mortality among patients with schizophrenia who live an average of 10-20 years less than the general population. Second generation antipsychotics are associated with obesity and other components of the metabolic syndrome.

**Objectives:**

The aim of this paper was to provide complete insight into the existing recent evidence for metabolic risks associated with the use of new antipsychotics, and establish recommendations for monitoring metabolic syndrome and other risks, as well as current options for treatment and prevention of metabolic syndrome.

**Methods:**

This review article is based on a literature search. We identified relevant publications and articles by searching the PUBMED database from 1999 to the present day according to the given parameters. The search criteria were the keywords “metabolic syndrome” combined with “schizophrenia” and “new antipsychotics”.

**Results:**

All researches has convincingly shown that patients with schizophrenia tend to be overweight and have a three to four times higher risk of developing diabetes than the general population. There are also more and more evidence in recent literature about the impact of new antipsychotics on the frequency of metabolic syndrome in patients with schizophrenia. The World Health Organization (WHO) defines metabolic syndrome as an elevated insulin level or a fasting glucose concentration of 5.6-6.0 mmol/l in combination with two or more of the following parameters: abdominal or central obesity and dyslipidemia and/or arterial hypertension. The research results systematically showed a 1.5 to 3 times higher frequency of metabolic syndrome in people suffering from schizophrenia compared to the general population. Therefore, regular control of all components of the metabolic syndrome is necessary, from waist circumference, which is the easiest to measure, to all others that can be carried out and done in the general practice doctor’s office.

**Conclusions:**

Metabolic changes in patients with shizophrenia who receive new antipsychotics in addition to their unfavorable lifestyle (improper diet, lack of physical activity, smoking) can lead to the development of metabolic syndrome and increase the risk for diabetes and cardiovascular diseases. It is therefore necessary to establish protocols for monitoring these risks and preventing comorbidities.

**Disclosure of Interest:**

None Declared

